# Analysis of Sports Supplement Consumption in 1688 Federated Road Cyclists

**DOI:** 10.3390/nu16010123

**Published:** 2023-12-29

**Authors:** Jesús García-Durán, José Antonio González-Jurado, Antonio Jesús Sánchez-Oliver

**Affiliations:** 1 Facultad de Ciencias del Deporte, Universidad Pablo de Olavide de Sevilla, 41013 Sevilla, Spain; 2 Departamento de Motricidad Humana y Rendimiento Deportivo, Universidad de Sevilla, 41013 Sevilla, Spain

**Keywords:** sports supplements, cycling, sport nutrition, ergogenic aids, performance, doping

## Abstract

The widespread use of sports supplements (SS) to enhance athletic performance extends to cyclists, although little research has been conducted on this subject within this sport. This descriptive and cross-sectional study involved 1688 federated road cyclists, aiming to analyse the pattern of SS consumption concerning the degree of scientific evidence and different categories. This study categorised SS based on the groups and subgroups established by the Australian Sport Institute (AIS, 2023) based on the level of evidence. Our results showed that 62.5% of the sample cyclists used SS, with an average of 12.2 ± 8.6 supplements consumed per participant. Health status (78.2%), pharmacies (62.5%), and medical doctors (45.7%) were the main reasons, purchase sites, and sources of information for SS consumption, respectively. The most prevalent SS consumed were Sports Gels (94%), Sports Bars (89.3%), and Sports Drinks (73.8%). Notably, 80% of the top ten most consumed SS belonged to the group with the highest level of evidence according to the AIS, with an average of 6.9 ± 3.2 supplements per participant. However, 23.3% of the total SS consumers used prohibited substances. In conclusion, while the prevalence of SS consumption among road cyclists is considerable and the primary sources for purchasing SS and obtaining advice are reliable, there is a notable prevalence of prohibited substance use within the sample.

## 1. Introduction

The popularity of road cycling has spread in recent years, with a global increase in the number of federation licenses, races, and events (Union Cycliste Internationale, 2023) [1]. In Spain, the competitive road cycling calendar typically spans from February to October, with competitions organised primarily by age categories and distances (Real Federación Española de Ciclismo, 2023) [2].

Cycling is a demanding sport [3], particularly from a metabolic perspective, as road cycling is characterised as an endurance sport with very high aerobic requirements. This is reflected in the elevated levels of maximum oxygen consumption and power output at the lactate threshold observed among competitive road cyclists under laboratory conditions [4]. However, it is essential to consider these factors in relation to anthropometric variables, given their influence on performance in this sport [4].

The performance of an individual cyclist is often attributed to factors such as maximum and functional power output, cardiovascular and pulmonary capacities, and other physiological abilities [5,6,7,8,9]. The extensively studied individual aspects include physiological, biomechanical, nutritional, aerodynamic, and physical components [5,6,7]. While these factors play a crucial role, overall sports performance in cycling is influenced by factors beyond the individual level. These include tactical characteristics emerging from interpersonal dynamics among cyclists, strategic elements related to the competition, and overarching features associated with the organisation of the sport [10].

Given the high demands of cycling and the potential for even marginal improvements to yield tangible benefits in performance and competition outcomes [11], it is imperative for cyclists to adhere to specific nutritional protocols tailored to their training or competition context [12]. The type, timing, and quantity of food consumed, along with the co-ingestion of ergogenic aids, are critical factors directly influencing athletic performance in these events [13,14,15,16].

Among the various tools and strategies utilised, sports supplements (SS) play a significant role. While there are numerous classifications of supplements based on the scientific evidence supporting them, one of the most widely adopted and current classifications is provided by the Australian Institute of Sport (AIS, 2023) [17]. The AIS categorises SS into four groups based on their level of scientific evidence and practical considerations related to safety, legality, and effectiveness in terms of enhancing athletic performance through the ABCD system. Group A includes supplements with a robust scientific foundation for improving athletes’ health and performance, which are further categorised into medical supplements, performance supplements, and Sports Foods (sub-groups). Group B comprises SS with potential benefits that require further research to confirm their effects. Group C consists of SS with evidence against their use. Lastly, Group D encompasses prohibited substances [17].

The use of SS in competitive sports is prevalent [18]. Their use varies based on factors such as the type of sport or event, age, competitive level, and gender [19]. Recent studies indicate substantial SS consumption in endurance sports, with higher usage observed at more competitive levels [20,21,22].

Cyclists stand out as significant consumers of SS, encompassing both dietary supplements and specialised sports foods [23]. These SS have the potential to serve as substantial sources of energy, macronutrients, micronutrients, and other nutritional elements within their overall intake during training or competition [12]. Moreover, the judicious use of specific SS can assist cyclists in achieving key objectives in sports nutrition and directly enhancing their performance in training or competitions [12]. This is a critical area that requires research, as SS, at their best, may contribute to attaining optimal nutrition and improving athletic performance. However, at their worst, they may lead to the wasting of money and pose risks of toxicity, along with unintended outcomes in doping tests [24].

Athletes often turn to SS with the anticipation of experiencing positive effects, primarily aiming to enhance their competitive performance [25]. However, many athletes lack the expertise to address critical questions before incorporating SS into their diet [14]. The first crucial consideration should be an evaluation of the available evidence regarding the safety of the supplement [26]. Following this step, an analysis of its efficacy becomes equally important, although athletes commonly prioritise these factors in reverse order [25]. Additionally, especially for professional and elite athletes, the potential risk of triggering a positive doping test should not be overlooked [27]. Athletes must have a valid rationale for using a particular supplement, requiring evidence indicating potential health or performance benefits [28]. This evidence should ideally apply to their specific sports context, as well as in the same sport at a comparable level [25]. It is unwise to assume that commercially available products are both effective and safe, as historical instances of food and supplement adulteration underscore the need for caution [29]. Even when evidence suggests potential benefits, individual responses may vary, and not every consumer is guaranteed to experience positive outcomes [13,14].

While some studies have addressed the consumption of sports supplements (SS) in cyclists [23,30,31,32], there is currently no exclusive research conducted on these athletes to investigate their prevalence and consumption patterns based on determining variables. Therefore, this study aims to analyse the SS consumption pattern among road cyclists, exploring potential differences based on categories and the level of evidence.

## 2. Materials and Methods

### 2.1. Type of Study

This is a quantitative cross-sectional and descriptive study focusing on the consumption of SS by road cyclists from the Andalusian Cycling Federation (FAC). The sample size was calculated using the Australian Bureau of Statistics’ (ABS) Sample Size Calculator (accessed on 25 November 2023). The confidence level was 95%. The size of the target population was 7818, according to data from the Andalusian Cycling Federation for the year 2022 (accessed on 18 July 2023). The expected proportion of the population was unknown; thus, 0.5 was assumed. Finally, 1668 cyclists participated in the study, with a confidence interval of 2.11%. The study population was selected through non-probabilistic sampling from within the FAC, cycling clubs, and sports associations in the Andalusian region.

### 2.2. Participants and Sample Size

A total of 1688 male federated cyclists participated in this study, all of whom were of legal age, with an average age of 36.4 ± 4.5 years. The distribution within the total sample included 150 subjects in the Sub-23 category, 258 subjects in the Elite category, 314 subjects in the Master-30 category, 342 subjects in the Master-40 category, 317 subjects in the Master-50 category, and 307 subjects in the Cyclotourist category. All participants were accustomed to training and competition, with no injuries or health conditions at the time of the survey. Table 1 provides information about the age, basic anthropometric characteristics, years of federation, weekly training days, hours of training per training day, and the number of competitions in the season for this study’s participants.

### 2.3. Instruments

A questionnaire that had previously been utilised in similar studies was administered [11,20,21,22,33,34,35]. The SS consumption questionnaire used underwent a prior validation process, which assessed its content, applicability, structure, and presentation [36]. The questionnaire comprised a total of 27 questions divided into three main sections: the first section gathered basic anthropometric and personal data (e.g., age, weight, height…), the second section focused on sports practice and its context (e.g., years of practice, number of competitions…), and the third and most extensive section was related to SS consumption. In this section, among other aspects, information was included about the types of supplements consumed, the reasons for their consumption, who provided advice on the matter, the places of acquisition, the timing of ingestion, perceptions regarding the results achieved after consumption, and doping. It is noteworthy that the questionnaire used in this study was one of 57 questionnaires (out of 164) reviewed, which were considered suitable for obtaining accurate information regarding athletes’ supplement use in the systematic review and meta-analysis conducted by Knapik et al. (2016) [18].

### 2.4. Procedure

The subject selection for this study was carried out by contacting the FAC and registered clubs and associations via email. They were provided with a cover letter and a document explaining this study’s characteristics and informed consent. Additionally, a link to the form was included to ensure that the participants obtained full information. Once this was performed, data were collected in person at various competitions held in the region from May to July 2023. Participation in this study was voluntary and anonymous. The protocol adhered at all times to the principles of the Declaration of Helsinki for human research, and it was approved by the ethics committee of Pablo de Olavide University (reference number: 23/7-3).

### 2.5. Statistical Analysis

Normal distribution and homoscedasticity were verified through the Kolmogorov–Smirnov and Levene tests. The data of the descriptive statistics are presented as mean (M) ± standard deviation (SD) for the quantitative variables and percentages and frequencies for the qualitative variables. Regarding the differential statistics, contingency tables were performed using the Chi-squared test to determine whether there were differences between categories. For pairwise comparisons between categories, a one-way ANOVA was performed when the assumptions of normality and homogeneity of variances were met; otherwise, a generalised linear model was conducted. In both cases, the Bonferroni adjustment was used for pairwise comparisons. The significance level was set at *p* < 0.05. The statistical analyses were carried out using the Statistical Package for Social Science v.20 for Windows (SPSS) (IBM. Armonk, NY, USA).

## 3. Results

### 3.1. General Aspects of SS Consumption

A total of 62.5% of the participants used SS (*n* = 1055) during the season in which they were surveyed. Dividing these results by categories, it was observed that 66% of Sub-23, 75.6% of Elite, 58% of Master-30, 51% of Master-40, 55.5% of Master-50, and 74.6% of Cyclotourists used SS. Significant differences (*p* < 0.001) were found between categories, including Sub-23 compared to Master-30 (*p* < 0.05); Elite compared to all other categories except Sub-23; Master-30 compared to Sub-23, Elite, and Cyclotourists; Master-40 compared to Elite; Master-50 compared to Elite and Cyclotourists; and Cyclotourists compared to Elite, Master-30, and Master-50.

### 3.2. Main Reasons for the Consumption of SS

**Figure 1:**
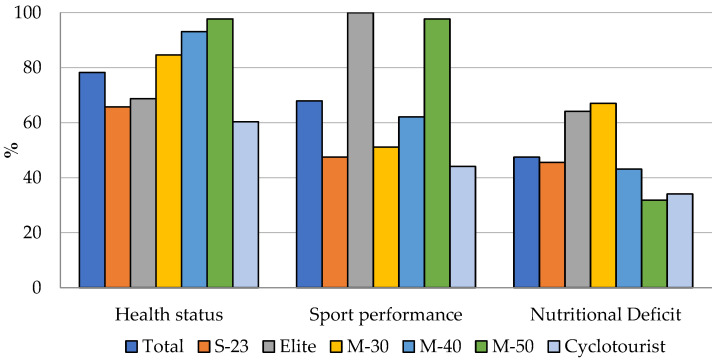
Most frequent reasons for SS use by total sample and each category.

Health status (78.2%), sport performance (67.9%), and nutritional deficit (47.5%) were the top three reasons for SS consumption selected by the total sample. Figure 1 illustrates the most frequent reasons for SS usage for the overall sample and each of the analysed categories.

It is worth noting that the Master-50, Elite, and Master-30 categories selected health status (97.7%), sport performance (100%), and nutritional deficit (67%), respectively, as the most frequent reasons for consuming SS. On the other hand, the least frequently selected reasons for SS consumption were health status (60.3%) and sport performance (44.1%) in the Cyclotourist category, as well as nutritional deficit (31.8%) in the Master-50 category.

In the pairwise comparisons of the most frequent reasons according to the studied categories, significant differences were observed, in at least one pairwise comparison, in all comparisons between categories (Supplementary Table S1), with Elite showing the largest number of differences and Sub-23 presenting the lowest number of differences. Regarding the three most frequent reasons, health status obtained the largest number of differences between categories, whereas nutritional deficit showed the lowest number of differences (Supplementary Table S1).

### 3.3. Purchase Locations for SS

**Figure 2:**
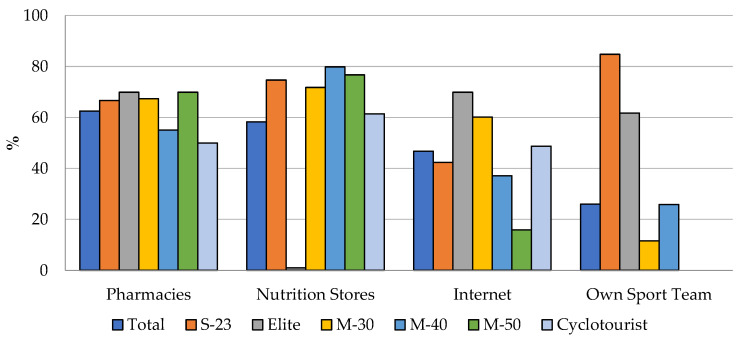
Main SS purchase location for the total sample and each category.

Pharmacies (62.5%), nutrition stores (58.3%), the Internet (46.8%), and a participant’s own sports team (26%) were the main SS purchase locations for the entire sample. Figure 2 presents the four most common SS purchase locations for the overall sample and each of the studied categories. The Sub-23 category (84.8%) showed the greatest consumption of SS provided by their own team. On the other hand, the Elite (1%), Master-50 (0%), and Cyclotourist (0%) categories presented the lowest consumption of SS provided by their own team.

The pairwise comparisons of the main SS purchase location according to the studied categories (Supplementary Table S2) produced significant differences in at least one pairwise comparison between categories in all comparisons, except for pharmacies in the Sub-23 category. In fact, this is the category with the lowest number of significant differences compared to the rest of the categories based on the most frequent purchase locations. On the contrary, the Elite category showed the largest number of differences. With regard to the main purchase locations for SS, it was observed that SS provided by their own team had the largest number of differences in pairwise comparisons between different categories. Conversely, pharmacies were the location with the lowest number of differences in pairwise comparisons (Supplementary Table S2).

### 3.4. Advisors or Sources of Information for SS Consumption

**Figure 3:**
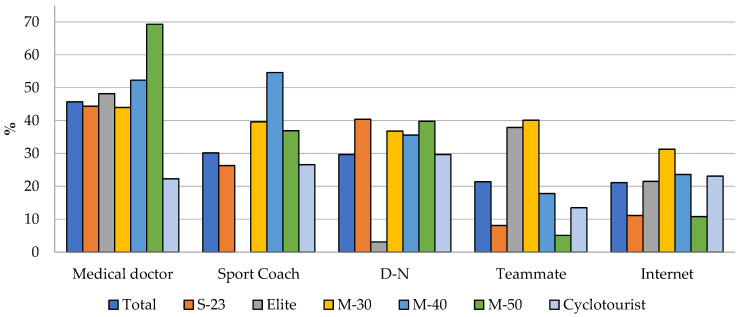
Sources of information when planning to use SS by total sample and each category.

Medical doctors (45.7%), sport coaches (30.2%), and Dietitian–Nutritionists (D-N) (29.7%) were the main advisors regarding SS consumption. Figure 3 illustrates the main advisors or sources of information for the consumption of SS for the total sample and each category. In this regard, the Master-50 category (52.3%) was the one with the largest number of participants who consulted medical doctors for advice on the use of SS. On the other hand, the Elite category was the one with the lowest number of participants who consulted sport coaches or D-N for this purpose.

The pairwise comparisons of main advisors or sources of information for SS use (Supplementary Table S3) showed significant differences in at least one pairwise comparison between categories in all comparisons, except for two cases: medical doctors, in the comparison of Master-30 with the other categories, and the Internet, in the comparison of Elite with the rest of the categories. The Sub-23 category presented the lowest number of significant differences compared to the rest of the categories regarding main advisors or sources of information for SS use.

On the other hand, the Elite category showed the largest number of differences compared to the other categories. D-N presented the lowest number of differences compared to the rest of the categories. Conversely, a participant’s own sports team was the advisor with the largest number of differences in pairwise comparisons between different categories (Supplementary Table S3).

### 3.5. Number of SS Consumed

The average number of SS consumed by the entire sample was 12.2 ± 8.6 SS. The category that consumed the largest number of SS was Master-30 (17.1 ± 9.6), followed by Elite (16.4 ± 10.3). On the other hand, the category with the lowest number of SS consumed was Cyclotourist (8.3 ± 6.1), followed by Master-50 (8.5 ± 4.1).

Table 2 shows the number of SS consumed based on the groups and subgroups of SS established by the AIS (2023) based on the level of evidence. It shows the number of SS consumed for the total sample and each of the analysed categories.

Regarding the categories, it is noteworthy that the Elite category had the largest number of differences in the number of SS consumed among the different groups and subgroups of SS by the level of evidence (AIS) compared to the rest of the categories. In contrast, the Sub-23 category, while presenting significant differences, had the lowest number of differences compared to the rest of the categories.

Observing the different groups and subgroups of SS by the level of evidence (AIS), a larger number of differences appeared in the total number of supplements consumed in groups A (total) and D, particularly in the Medical Supplements subgroup (Group A). On the contrary, although significant differences can be observed, the groups with the lowest number of differences were Group B and the Performance Supplements subgroup (Group A).

A total of 23.3% of the participants used prohibited substances. Finally, Table 3 shows the percentage of Group D supplements consumed by the entire sample and each of the analysed categories. 

### 3.6. Most Consumed SS

**Figure 4:**
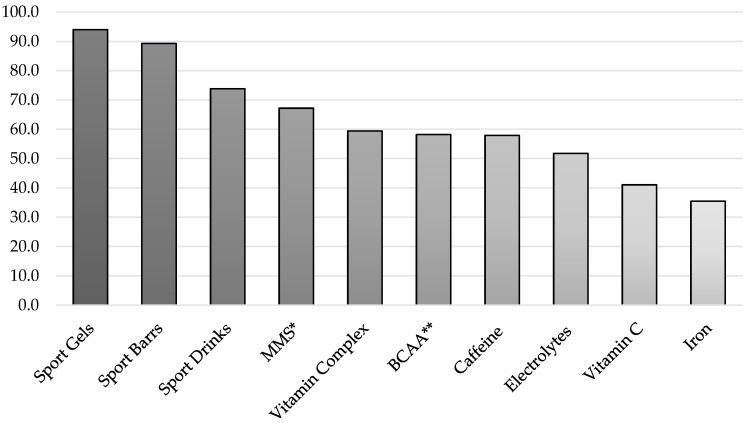
Top ten SS used by the total sample. * MMS: Mixed macronutrient supplement; ** BCAA: branched-chain amino acids.

Figure 4 shows the top ten SS most used by the total sample. The five most consumed SS by the sample were Sports Gels (94%), Sports Bars (89.3%), Sport Drinks (73.8%), mixed macronutrient supplements (67.2%), and multivitamins (59.4%). All of these belong to the group with the highest level of evidence according to the AIS (Group A), with the first four belonging to the Sport Foods subgroup. BCAA (58.2%) and Caffeine (57.9%), from Group C and Group A (Performance Supplements subgroup), respectively, complete the list of the SS most commonly consumed by the entire sample. These seven SS, although in different orders and proportions, are part of the top five most used in each of the analysed categories (Table 3).

The pairwise comparisons between categories, as reflected in Table 4, showed numerous significant differences (*p* < 0.05) among the most consumed SS. Regarding the categories, it can be observed that the Sub-23 and Cyclotourist categories had the largest number of differences compared to the rest of the categories. On the contrary, the Master-50 category had the lowest number of differences compared to the rest of the categories.

Focusing on the top ten most consumed SS, significant differences were observed in all SS when comparing them across different categories, with BCAA being the SS with the largest number of differences. In contrast, Sports Gels was the SS with the lowest number of differences when SS were compared across different categories (Table 4).

## 4. Discussion

The aim of the present study was to examine the patterns of SS consumption among road cyclists, with a focus on understanding prevalence and consumption patterns based on determining variables. While previous studies have explored SS consumption in cyclists [23,30,31,32], there is currently no exclusive research on these athletes that investigates their prevalence and consumption patterns via consideration of specific variables and divided into different categories.

### 4.1. General Aspects of SS Consumption

A total of 62.5% of the sample in the current study used SS. These data are considerably lower than those reported in Canadian Olympic cyclists (100%) during the Sydney Olympics [31], elite U-23 male cyclists (97.5%) [23], and high-level Spanish cyclists (85%) [32]. This may be due to the difference in the level of competition, since the level of performance or competition of the athlete is one of the main determinants of SS consumption [37]. This is supported by the results obtained by dividing the sample into categories, with the Elite category showing the greatest use of SS (75.6%) with respect to the other categories. However, SS consumption is high compared to those in other studies carried out in other sports [18]. Additionally, several studies have confirmed that endurance athletes appear to consume more supplements [38,39].

### 4.2. Main Reasons for the Consumption of SS

Athletes who engage in prolonged, intense physical activity typically report that their primary reason for using SS is to improve performance, although improving/maintaining health may also be an important reason [20,21,22,32,40]. These data are similar to those found in the present study, although they are in a different order. These results may be due to the age difference between our study (36.4 ± 4.5 years) and a different study of SS consumption in cyclists (20.7 ± 1.3 years) [23]. These outcomes differ from those of several studies of other endurance sports disciplines with similarly aged participants (from 34.8 to 43.1 years) [20,21,22], in which “sport performance” was the most selected reason (from 47.4% to 82.3%) [20,21,22]. This finding is supported by the data found when dividing the sample by category, with the Master-50 (97.7%) and Master-40 (93.1%) categories selecting “health status” as the first reason. On the contrary, the Elite category (100%), which dedicates the largest number of days per week (4.8 ± 0.8) and the largest number of hours per day (5.3 ± 0.9) to training, marked “sport performance” as the first reason. “Nutritional deficit” (47.5%) was the third most frequently chosen reason. These data coincide with those reported in a similar study of elite U-23 male cyclists, where 49.1% indicated “to integrate a dietary deficiency” as the main reason [23].

### 4.3. Purchase Locations for SS

The SS purchase location can be a determining factor for its correct use [25], since it can contribute to better advice and the purchase of higher-quality products [26]. The first two options chosen by the sample, i.e., pharmacies and specialised stores, can help with this issue, reducing the possibility of the contamination of SS with prohibited substances compared to online purchases [25,41]. Although not in this order, these sites are the most frequent in studies of other endurance sports disciplines [20,21,22]. These data are in disagreement with those reported in elite Spanish athletes of different disciplines, in which only approximately 1% went to the pharmacy to buy SS [32]. This finding is related to the first purpose chosen by the sample for SS consumption in the present study, i.e., health status.

Similarly, the Internet is often reported as a site of SS purchasing preference [33,42]. This finding indicates that a high frequency of the surveyed cyclists may be at risk of involuntary doping due to their behaviour when purchasing SS [43,44]. The global sports supplement industry has experienced significant growth, which is expected to reach USD 230.7 billion by 2027, driven by heightened awareness of aesthetics, health, and sport performance. However, this growth poses risks for consumers, including elite and recreational athletes [45]. Geller et al. (2015) report approximately 23,000 annual emergency room visits due to adverse events related to sports supplements [46]. The lack of global regulation on dietary supplements, the risk of contamination, and insufficient information on proper use and scientific basis increase the likelihood of inadequate or excessive supplement consumption or unintentional doping, particularly in the unregulated online market [41]. Some studies highlight concerns about online purchases, where sports supplements may contain undisclosed substances, incorrect doses, or other contaminants, risking athletes’ health, performance, and careers [28,45,47].

### 4.4. Advisors or Sources of Information for SS Consumption

It is worth highlighting the most frequently consulted professionals for advice on SS in the present study. This finding is in disagreement with the results reported in other studies, where the sources of information used by athletes are of poor quality, such as family members, teammates, friends, and even the Internet [23,48,49]. Nevertheless, this finding is in line with the results reported in a similar study, in which medical doctors were the main sources of information used (74.4%) by cyclists [23]. Moreover, and although not in the same order, the main sources of information for the use of SS coincide with the data reported in studies of other endurance sports disciplines [20,21,22]. Thus, the role of D-N is becoming more important, since the latest studies on SS consumption highlight them as the first or second advisors for athletes [20,21,22]. In this respect, athletes who receive information from a D-N as the main source of nutritional information have better eating habits, a better understanding of the nutrient timing, and greater scientific evidence to inform the choice of SS [50]. In the comparisons by category, it is worth highlighting the Elite category, which presented the largest number of differences compared to the rest, emphasising that it is the one that least frequently consults the D-N (3.1%). This shows the need to focus on the correct sources of advice, as bad information and bad advice can lead to the consumption of non-evidence-based SS or, even worse, to a case of unintentional doping and health risk [28,47].

### 4.5. Number of SS Consumed

The average number of SS consumed by the sample was well above the values recently reported in mountain runners (6.9) [20], triathletes (8.3) [21], open water swimmers (4.67) [21], and elite athletes from different sports disciplines (3.0) [32]. The type of sport is another SS consumption variable, with some sports having a greater use of SS than others [18]. In the case of cycling, this is reflected in a study in which SS consumption was compared in several sports performed at two Olympic games; in this study, cycling was the sport with the highest prevalence of consumption compared with the rest [31]. As was previously noted, one of the greatest determining variables in terms of consumption is the level of competition [13,14]. This is reflected again when dividing the sample into categories, with the Elite category being the one that consumed the largest number of SS and Cyclotourists consuming the lowest number of SS, with significant differences between them.

Regarding the type of SS in relation to the level of evidence [17], it is worth highlighting the large number of SS consumed in Group A compared to the rest of the groups. These results are related to the aforementioned aspects: place of purchase and source of information. Without a doubt, this is a very positive finding. Furthermore, this trend is supported by similar current studies in which SS of Group A were the most frequently consumed [20,21,22]. The scientific dissemination on SS is having an impact on the target population and end consumers. Additionally, current evidence suggests that a nutrition education intervention can positively influence the sports nutrition knowledge of athletes [51], and, thus, it could be useful.

Within Group A, there was a notable difference between the number of SS used in the Sport Foods subgroup and those of the other two subgroups. Again, these results are supported by data found in recent publications in which athletes from different endurance sports were surveyed [20,21,22]. The characteristics of training and competitions and the weather conditions in cycling can lead to the inclusion of SS from the Sports Foods subgroup (Groups A), such as Sports Drinks, Sports Gels, sports confectionery, Sports Bars, electrolyte supplements, isolate protein supplements, and mixed macronutrient supplements [17]. The inclusion of these is essential in this sport, since they can help to control the appearance of performance-limiting factors or prevent adverse health situations [6,7], such as restoring hydroelectrolyte balance and preventing dehydration; preventing the deterioration of endurance, strength, blood volume, and cognitive function in competitions of this duration; helping to maintain and replenish muscle glycogen; reducing the muscle damage produced; and improving post-exercise recovery [8,10,12,52].

On the other hand, a high number of SS is found to be consumed in Group C, i.e., the second most frequent. SS with evidence against their use belong to this group. As in the present study, this SS group was the second most consumed after Group A in recent studies [20,21,22]. The possible negative effects associated with the use of these SS range from a positive doping test to deterioration in performance and adverse health effects [25]. In addition, competitive athletes should also be concerned about overuse and possible adverse interactions due to polypharmacy [53] and inadvertent doping due to inadequate quality control of some dietary supplements such as those in Group C [54]. A better-educated athlete would probably consume a lower number of supplements promoted by the dietary supplement lobby [32,45,55].

### 4.6. Most Frequently Consumed SS

The results regarding the most frequently consumed supplements reveal that all the SS in the top ten belong to Group A of the AIS [17], except for branched-chain amino acids (BCAA) and Vitamin C, which belong to Group C and B, respectively. While this is a positive aspect to highlight, it is worth noting that some of the SS with scientific evidence, such as sodium bicarbonate (NaHCO_3_) and glycerol, are not among the twenty most consumed SS. Recent studies support the use of NaHCO_3_ in endurance sports [56]. One of them examined the effect of stacked NaHCO_3_ loading before and during a 3-h endurance cycling test of final-stage all-out performance. Administering 300 mg/kg of NaHCO_3_ enhanced blood HCO_3_ levels, leading to improved power output in a 90-second exercise bout [57]. Another study found that 0.3 g·kg^−1^ BW of NaHCO_3_ was effective in improving performance, presenting higher blood lactate levels in cyclists associated with significant improvements in pH levels [58]. On the other hand, beverages containing glycerol create an osmotic gradient in circulation, promoting fluid retention, thus facilitating hyperhydration and protecting against dehydration [59]. In doing so, hyperhydration with glycerol could be a useful method due to the simultaneous decrease in urine production and elimination of free water, providing a performance advantage by offsetting dehydration [60], which is crucial in road cycling [61]. Understanding these aspects or others about SS with evidence levels can assist various stakeholders in making better decisions about SS use by road cyclists.

When comparing sports supplements (SS) across different categories, Sports Gels stood out as the least varied SS, and it was the most consumed SS in all categories except for Elite, where it was the second most frequently consumed SS. This aligns with findings of similar studies among endurance athletes, where sports gels, energy bars, and sports drinks are among the top three consumed SS [20,21,22].

Although the information sources were reliable, the purchase location was appropriate, and there was high consumption of Group A sports supplements (SS) among the top 20 most consumed, as well as several SS from Group C, which have evidence against their use. This pattern is consistent with findings reported in several recent studies [19,24,35]. BCAA, Spirulina, and Glutamine are some of these Group C SS. The indiscriminate use of supplements raises concerns and calls for educational interventions from an early age for athletes, coaches, and parents/family members [43]. It is essential to emphasise that the use of dietary supplements should not replace healthy food choices and a proper diet, except as a temporary strategy when dietary changes are not feasible [14]. Professionals and athletes must recognise that a well-planned diet supports the benefits of evidence-based supplements for enhancing performance, delaying fatigue, altering body composition, and improving health [62].

Particular attention should be paid to the consumption of Group D supplements by the sample, as 23.3% of the total SS consumers were using these prohibited substances [17]. This prevalence increased in the Elite category (33.3%). Despite the limitations in the attainment of precise data regarding the prevalence of doping agent use, studies suggest that the illicit use of these agents, among both athletes and non-athletes, can range from 1% to 5% in the general population, exceeding 50% in certain groups, with higher prevalence in men [27]. The use of doping agents, such as anabolic steroids, stimulants, and erythropoietin, increases the risk of cardiovascular disease, thrombosis, stroke, and cancer in both genders. Anabolic steroids negatively impact hormonal axes, suppressing hormone production in men and inducing virilising effects in women. Regular use of these agents can also have serious consequences on mental health and behaviour. Therefore, the health risks associated with using these agents may outweigh the physical and mental benefits of physical activity [27].

The literature on doping in sports includes studies on its prevalence; measurement methods; incidence among youth, enthusiasts, and professionals; its presence in specific sports, and the types of substances or supplements consumed [63]. In elite cycling, the use of prohibited substances is widespread [27,64]. This issue is extending to young and amateur cyclists [65,66]. Several studies have demonstrated a connection between elite and amateur athletes who use supplements and intentional doping [67,68]. The use of supplements can serve as a “gateway to doping”, with doping rates being 3.5 times higher in the group using dietary supplements compared to those who do not use supplements [69].

### 4.7. Limitations, and Future Perspectives

While our study presents novel findings and includes a substantial sample of federate road cyclists, it is important to acknowledge several limitations. One notable constraint is that the conclusions should be interpreted cautiously due to the reliance on self-reported data, which may introduce errors. Additionally, this study only considered supplements consumed during the specific season in which the data were collected, omitting potential long-term effects of supplement use on athletes’ health. These limitations underscore the need for careful consideration when concluding the study.

It is essential for athletes and their professional advisors to use supplements appropriately and responsibly. Understanding nutritional substances is crucial due to their relevance for both performance and athlete health. This knowledge should encompass not only the effectiveness of supplements but also their safety and compliance with legal regulations, as these three aspects (effectiveness, safety, and legality) are the primary sources of issues related to supplements in a sporting context [25,26,38].

## 5. Conclusions

The prevalence of sports supplement (SS) consumption among road cyclists is significant. The primary sources for purchasing and obtaining advice on SS are trustworthy. While the most frequently consumed SS are backed by scientific evidence, there is a notable prevalence of prohibited substance use within the sample. Although the overall results concerning the sports supplement consumption pattern were favourable, the elevated intake of prohibited supplements by the sample poses a health risk and increases the likelihood of a positive outcome in doping tests. This underscores the need for additional education on this matter.

## Figures and Tables

**Table 1:** Main participant characteristics.

Category	Age (Years)	Height (cm)	Weight (kg)	Years Licensed	Weekly Training Days	Daily Training Hours	N° Competitions in the Season
Total (*n* = 1688)	36.4 ± 4.5	172.1 ± 6.8	65.5 ± 7.3	6.4 ± 2.6	4.5 ± 0.8	4.3 ± 0.8	7.8 ± 0.1
Sub-23 (*n* = 150)	20.5± 1.1	173.5 ± 7.1	64.0 ± 6.8	4.7 ± 2.2	4.7 ± 0.6	4.8 ± 0.9	5.3 ± 0.1
Elite (*n* = 258)	29.9 ± 4.1	172.4 ± 6.6	62.6 ± 6.7	5.9 ± 2.8	4.8 ± 0.8	5.3 ± 0.9	6.3 ± 0.1
Master-30 (*n* = 314)	34.6 ± 3.0	172.7 ± 6.9	64.9 ± 7.9	5.3 ± 2.8	4.5 ± 0.8	4.4 ± 0.8	7.3 ± 0.1
Master-40 (*n* = 342)	44.6 ± 2.9	173.4 ± 7.1	67.7 ± 7.4	7.1 ± 3.2	4.3 ± 0.8	3.9 ± 1.0	8.3 ± 0.1
Master-50 (*n* = 317)	54.6 ± 2.8	166.1 ± 5.8	64.2 ± 5.4	9.5 ± 1.5	4.0 ± 0.7	3.3 ± 0.9	9.3 ± 0.1
Cyclotourist (*n* = 307)	34.4 ± 13.6	174.8 ±7.6	69.8 ± 9.4	6.1 ± 2.9	4.5 ± 1.1	4.3 ± 0.1	10.3 ± 0.1

Categories distributed by the International Cycling Union (UCI). Data presented as M ± SD.

**Table 2:** Number of SS consumed by the total sample and each of the categories, divided by SS groups according to the level of evidence of the AIS (2023).

SS Group by Level of Evidence (AIS)		Total (*n* = 1055)	S-23 (*n* = 99)	Elite (*n* = 195)	M-30 (*n* = 182)	M-40 (*n* = 174)	M-50 (*n* = 176)	Cyclotourist (*n* = 229)
Group A	Sports Foods	4.2 ± 1.5	3.6 ± 2.2 ^ab^	5.6 ± 1.7 ^acdef^	4.1 ± 1.1 ^bcg^	4.4 ± 1.4 ^dh^	4.1 ± 0.9 ^ei^	3.2 ± 2.0 ^fghi^
	Medical Supplement	0.7 + 1.7	0.7 ± 0.1 ^abcd^	1.9 ± 1.3 ^aefg^	1.6 ± 1.1 ^bhij^	1.3 ± 1.2 ^ceh^	1.3 ± 1.1 ^dfik^	0.9 ± 1.1 ^gjk^
	Performance Supplement	1.5 ± 1.2	1.3 ± 1.2 ^a^	1.9 ± 1.5 ^abc^	1.6 ± 1.3 ^de^	1.7 ± 1.3 ^fg^	0.9 ± 0.7 ^bdf^	1.0 ± 1.4 ^ceg^
	Total of Group A	6.9 ± 3.2	5.6 ± 4.0 ^abc^	9.4 ± 4.1 ^adefg^	7.5 ± 2.9 ^bdhi^	7.4 ± 3.0 ^cejk^	6.2 ± 2.1 ^fhjl^	5.2 ± 3.8 ^gikl^
Group B		0.9 ± 0.9	1.2 ± 0.9 ^abc^	1.3 ± 1.2 ^defg^	0.7 ± 1.1 ^d^	0.8 ± 0.8 ^e^	0.7 ± 0.8 ^f^	0.6 ± 0.9 ^g^
Group C		3.4 ± 3.7	3.8 ± 4.3 ^a^	4.9 ± 4.8 ^bcd^	4.2 ± 4.7 ^fg^	3.2 ± 3.3 ^h^	1.6 ± 1.7 ^acfhi^	2.9 ± 3.7 ^dgi^
Group D		0.3 ± 0.7	0.3 ± 0.7 ^ab^	0.7 ± 1.2 ^acdef^	0.4 ± 0.9 ^cgh^	0.3 ± 0.7 ^di^	0.1 ± 0.1 ^begij^	0.1 ± 0.5 ^afhj^
Total of Supplements		12.2 ± 8.6	10.9 ± 8.9 ^a^	16.4 ± 10.3 ^abcde^	12.8 ± 8.9 ^bfg^	11.8 ± 6.9 ^chi^	8.5 ± 4.1 ^dfh^	8.9 ± 7.9 ^egi^

Data presented as M ± SD. Differences between categories using a generalised linear model. The same superscript (letters) indicates differences between pairs (Bonferroni *p* < 0.05).

**Table 3:** Percentage of Group-D SS consumed by the total sample and each category of Group D.

	Total	S-23	Elite	M-30	M-40	M-50	Cyclotourist
	(*n* = 1688)	(*n* = 150)	(*n* = 258)	(*n* = 314)	(*n* = 342)	(*n* = 317)	(*n* = 307)
Yes	23.3	27.3	33.3	22.9	21.1	21.5	17.6
No	75.1	72.7	66.7	76.4	76.0	77.0	78.8
DK/NR	1.7	0.0	0.0	0.6	2.9	1.6	3.6

**Table 4:** Most consumed SS for the total sample and each category.

Supplements	Total (*n* = 1055)	S-23 (*n* = 99)	Elite (*n* = 195)	M-30 (*n* = 182)	M-40 (*n* = 174)	M-50 (*n* = 176)	Cyclotourist (*n* = 229)
Sports Gels	94	96 ^a^	95.9 ^b^	99.5 ^c^	99.4 ^d^	99.4 ^e^	78.5 ^abcde^
Sports Bars	89.3	64.6 ^abcde^	99.5 ^af^	96.7 ^bg^	98.3 ^ch^	97.2 ^di^	72.8 ^efghi^
Sports Drinks	73.8	51.5 ^abcde^	92.8 ^afgh^	92.3 ^bijk^	65.5 ^cfilm^	78.4 ^dgjln^	55.7 ^ehkmn^
MMS *	67.2	61.6 ^abcde^	85.6 ^afgh^	43.4 ^bfijk^	76.4 ^cgilm^	87.5 ^djln^	50.4 ^ehkmn^
Vitamin Complex	59.4	20.2 ^abcde^	85.6 ^afghi^	91.8 ^bfjkl^	27 ^cgjmn^	61.9 ^dhkm^	51.3 ^eiln^
BCAA **	58.2	54.5 ^abcde^	75.4 ^afghi^	70.9 ^bfjkl^	59.2 ^cgjmn^	48.3 ^dhkmo^	42.1 ^eilno^
Caffeine	57.9	55.6 ^abcd^	68.7 ^aefg^	61 ^behi^	71.8 ^chjk^	59.1 ^fjl^	36 ^dgikl^
Electrolytes	51.8	45 ^abcde^	79.5 ^afghi^	58.2 ^bfjk^	54.6 ^cglm^	34.7 ^dhjl^	36.8 ^eikm^
Vitamin C	41	36.4 ^abcde^	62.1 ^afghi^	22.5 ^bfjkl^	52.9 ^cgjm^	44.9 ^dhk^	28.1 ^eilm^
Iron	35.5	29.3 ^abcde^	43.1 ^afg^	40.7 ^bh^	38.5 ^ci^	40.3 ^dj^	21.5 ^eghij^
Nitrate	28.6	37.4 ^abc^	42.1 ^adef^	41.2 ^ghi^	33.9 ^dgjk^	6.3 ^behjl^	16.7 ^cfikl^
Hydrolysate Casein	25.5	28 ^abcde^	34.9 ^afgh^	40.1 ^bfijk^	33.3 ^cilm^	5.7 ^dgjln^	14 ^ehkmn^
Beta-Alanine	24.2	32.3 ^abcd^	41.5 ^aefgh^	30.2 ^beijk^	24.1 ^film^	2.8 ^cgjln^	17.5 ^dhkmn^
Probiotics	23.9	21.2 ^abcd^	34.9 ^aefgh^	25.8 ^beij^	13.8 ^cfik^	29.5 ^dgkl^	17.5 ^hjl^
Creatine Monohydrate	23.5	0 ^abcde^	32.8 ^afghi^	20.3 ^bfj^	40.2 ^cgjkl^	17.6 ^dhk^	20.2 ^eil^
Mineral Complex	22.5	26.3 ^abcdh^	39.5 ^aefg^	28.6 ^eijk^	21.3 ^bfilm^	8.5 ^cgjln^	13.2 ^dhkmn^
Spirulina	22.2	26.3 ^abcd^	27.7 ^efgh^	33.5 ^aeij^	34.5 ^bfkl^	9.7 ^cgik^	7 ^dhjl^
Melatonin	20.9	18.2 ^abcd^	23.6 ^aef^	19.8 ^gh^	28.2 ^begij^	23.9 ^cik^	12.7 ^dfhjk^
Citrulline Malate	19.5	0 ^abcde^	33.8 ^afgh^	23.1 ^bfgijk^	30.5 ^cilm^	8 ^djl^	13.6 ^ehkm^
Glutamine	18.8	31 ^abcde^	23.1 ^afgh^	17 ^bfij^	24.7 ^cikl^	10.2 ^dgk^	13.2 ^ehjl^

Data presented as %. Differences between categories using a generalised linear model. The same superscript (letters) indicates differences in pairs (Bonferroni *p* < 0.05). * MMS: Mixed macronutrient supplement; ** BCAA: branched-chain amino acids.

## Data Availability

The data are stored in the research data repository of the University of Seville—https://doi.org/10.12795/11441/152413.

## Supplementary Materials

The following supporting information can be downloaded at: https://www.mdpi.com/article/10.3390/nu16010123/s1, Table S1. Main consumer purposes for SS consumption by total sample and each category. Table S2. Most common place of purchase of SS by total sample and each category. Table S3. Main advisors or sources of information about the consumption of SS by total sample and each category.
