# Supplementary material for: Analysis of Sports Supplement Consumption in 1688 Federated Road Cyclists
Source: Nutrients. 2023 Dec 29;16(1):123. doi: 10.3390/nu16010123 (PMC10780486; doi:10.3390/nu16010123)
Supplement: Supplementary file 1 [file nutrients-16-00123-s001.zip › nutrients-2778999-supplementary.pdf]

Supplementary Table S1. Main consumer purposes for SS consumption by total sample and each category.

	Total (n=1055)	S-23 (n=99)	Elite (n=195)	M-30 (n=182)	M-40 (n=174)	M-50 (n=176)	Cyclotourist (n=229)
**Health Status**	78.2	65.7^{abc}	68.7^{def}	84.6^{adgh}	93.1^{be}	97.7^{cfi}	60.3^{ghi}
**Sport Performance**	67.9	47.5^{ab}	100.0^{acde}	51.1^{cf}	62.1^{dg}	97.7^{bfi}	44.1^{egi}
**Nutritional Deficit**	47.5	45.5^{a}	64.1^{bcd}	67.0^{aef}	43.1^{be}	31.8^{cf}	34.1^{d}

Data presented as %. Differences between categories using a generalized linear model. The same superscript (letters) indicates differences in pairs (Bonferroni $p<0.05$).

Supplementary Table S2. Most common place of purchase of SS by total sample and each category.

	Total (n=1055)	S-23 (n=99)	Elite (n=195)	M-30 (n=182)	M-40 (n=174)	M-50 (n=176)	Cyclotourist (n=229)
**Pharmacies**	62.5	66.7	69.9^{ab}	67.4^{c}	55.1^{a}	69.9^{d}	50.0^{bcd}
**Nutrition Stores**	58.3	74.7^{a}	1.0^{abcde}	71.8^{b}	79.8^{cf}	76.7^{dg}	61.4^{efg}
**Internet**	46.8	42.4^{abc}	69.9^{adef}	60.2^{bgh}	37.1^{dgi}	15.9^{cehij}	48.7^{fj}
**Own Sport Team**	26.0	84.8^{abcde}	61.7^{afghi}	11.6^{bfjkl}	25.8^{cgjmn}	0.0^{dhkmo}	0.0^{eilno}

Data presented as %. Differences between categories using a generalized linear model. The same superscript (letters) indicates differences in pairs (Bonferroni $p<0.05$).

Supplementary Table S3. Main advisors or sources of information about the consumption of SS by total sample and each category.

	Total (n=1055)	S-23 (n=99)	Elite (n=195)	M-30 (n=182)	M-40 (n=174)	M-50 (n=176)	Cyclotourist (n=229)
**Medical doctor**	45.7	44.4^{ab}	48.2^{cd}	44	52.3^{e}	69.3^{acf}	22.3^{bdef}
**Sport Coach**	30.2	26.3^{ab}	0^{acdef}	39.6^{bcg}	54.6^{dhi}	36.9^{eh}	26.6^{fgi}
**D-N**	29.7	40.4^{a}	3.1^{abcde}	36.8^{b}	35.6^{c}	39.8^{d}	29.7^{e}
**Teammate**	21.4	8.1^{ab}	37.9^{acde}	40.1^{bfgh}	17.8^{cfi}	5.1^{dgij}	13.5^{ehj}
**Internet**	21.1	11.1^{a}	21.5	31.3^{ab}	23.6^{c}	10.8^{bcd}	23.1^{d}

D-N: Dietitian-Nutritionist. Data presented as %. Differences between categories using a generalized linear model. The same superscript (letters) indicates differences in pairs (Bonferroni $p<0.05$).
